# Effects of Sorafenib and Arsenic Trioxide on *U937* and KG-1 Cell Lines: Apoptosis or Autophagy?

**DOI:** 10.22074/cellj.2020.6728

**Published:** 2019-12-15

**Authors:** Atousa Haghi, Mahdieh Salami, Mahnaz Mohammadi Kian, Mohsen Nikbakht, Saeed Mohammadi, Bahram Chahardouli, Shaharbano Rostami, Kianoosh Malekzadeh

**Affiliations:** 1. Hematology, Oncology and Stem Cell Transplantation Research Center, Tehran University of Medical Sciences, Tehran, Iran; 2. Young Researchers and Elite Club, Tehran Medical Sciences, Islamic Azad University, Tehran, Iran; 3. Hematologic Malignancies Research Center, Tehran University of Medical Sciences, Tehran, Iran; 4. Molecular Medicine Research Center (MMRC), Hormozgan University of Medical Science (HUMS), Bandar Abbass, Iran

**Keywords:** Acute Myeloid Leukemia, Apoptosis, Arsenic Trioxide, Cell Proliferation, Sorafenib

## Abstract

**Objective:**

Acute myeloid leukemia (AML) is a clonal disorder of hemopoietic progenitor cells. The Raf serine/threonine (Ser/Thr) protein kinase isoforms including B-Raf and RAF1, are the upstream in the MAPK cascade that play essential functions in regulating cellular proliferation and survival. Activated autophagy-related genes have a dual role in both cell death and cell survival in cancer cells. The cytotoxic activities of arsenic trioxide (ATO) were widely assessed in many cancers. Sorafenib is known as a multikinase inhibitor which acts through suppression of Ser/Thr kinase Raf that was reported to have a key role in tumor cell signaling, proliferation, and angiogenesis. In this study, we examined the combination effect of ATO and sorafenib in AML cell lines.

**Materials and Methods:**

In this experimental study, we studied *in vitro* effects of ATO and sorafenib on human leukemia cell lines. The effective concentrations of compounds were determined by MTT assay in both single and combination treatments. Apoptosis was evaluated by annexin-V FITC staining. Finally, mRNA levels of apoptotic and autophagy genes were evaluated using real-time polymerase chain reaction (PCR).

**Results:**

Data demonstrated that sorafenib, ATO, and their combination significantly increase the number of apoptotic cells. We found that the combination of ATO and sorafenib significantly reduces the viability of U937 and KG-1 cells. The expression level of selective autophagy genes, *ULK1* and *Beclin1* decreased but LC3-II increased in U937.

**Conclusion:**

The expression levels of apoptotic and autophagy activator genes were increased in response to treatment. The crosstalk between apoptosis and autophagy is a complicated mechanism and further investigations seem to be necessary.

## Introduction

Acute myeloid leukemia (AML) as a malignant disease of the bone marrow, is caused by acquired somatic mutations and chromosomal rearrangements which occur in a hematopoietic progenitor. Regardless of its etiology, the AML pathogenesis involves extraordinary differentiation and proliferation of a clonal population of myeloid stem cells. Different processes involved in leukemia are controlled by signaling pathways initiated by activated receptor tyrosine kinases (RTKs) (1).

RAS is a downstream factor for various RTKs. Activation of RAS signaling pathway has a critical function in the development of human malignancies (2). Fundamental activity of the RAS pathways arises from downstream effectors of RAS, activating mutations in the RAS, or even overexpression of a variety of RTKs, including vascular endothelial growth factor receptors (VEGFRs), epidermal growth factor receptor (EGFR) or platelet-derived growth factor receptor (PDGFR) (3). Therefore, RAS mutations or activation in human tumors could lead to cell survival and proliferation. RAS adjusts multiple pathways such as RAF/MEK/ERK pathway which remarkably activate cellular transformation

RAF kinases are serine/threonine protein kinases which act as a downstream effector of RAS. The Raf serine/ threonine protein kinase isoforms including A-Raf, B-Raf and Raf1, are the upstream in the MAPK cascade (4) and they regulate cellular proliferation and survival. Moreover, it was recently demonstrated that wild-type Raf1 could, independently of MAPK signaling, promote cell survival, through interactions with apoptosis and antiapoptosis regulatory proteins (5).

Beclin1 (which is encoded by *BCNG 1* gene) is one of the core autophagy-regulating elements and a haploinsufficient tumor suppressor gene which is directly associated with BCL-2 (6). ULK1 is a serine/threonineprotein kinase that is involved in autophagy pathways (7). LC3 (an ubiquitin-like protein) is a soluble protein that is distributed in cultured cells and tissues. During autophagy activation, LC3-I is found in the cytoplasm and it is also conjugated with phosphatidylethanolamine via LC3-phosphatidylethanolamine conjugate (LC3-II) that induces formation and elongation of the autophagosome (8). PTEN as a tumor suppressor is one of the most commonly deleted, mutated or promoter methylated genes in various cancers. PTEN is able to control autophagy based upon lipid phosphatase activity that opposes the function of PI3K and also deactivates Akt and mTOR signaling (9).

**Fig 1:**
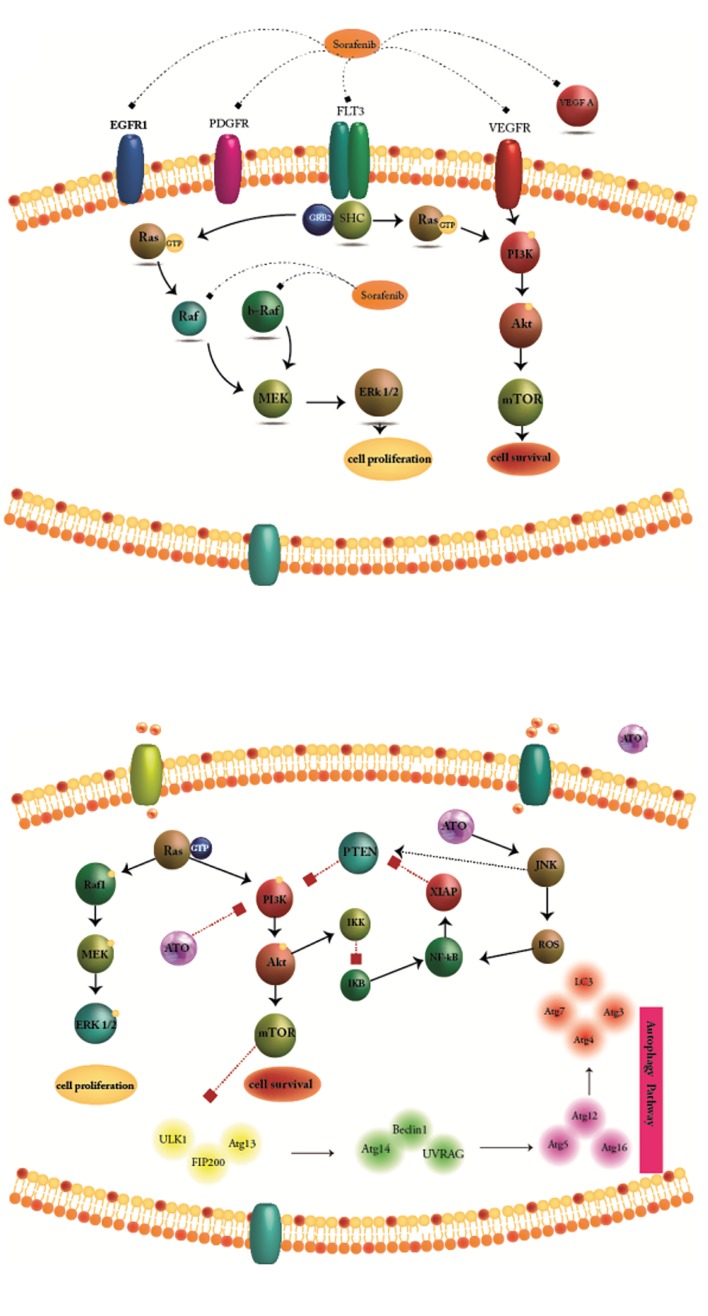
Molecular target of sorafenib and arsenic trioxide (ATO). **A.** Sorafenib is known as a multikinase inhibitor which acts through suppressing Ser/ Thr kinase Raf that is known to have important roles in tumor cell signaling and proliferation and **B.** ATO as a single agent, targets various cellular functions through affecting multiple molecular factors. ATO activates both autophagy and apoptosis.

Sorafenib is known as a multikinase inhibitor which has effective roles in tumor cell signaling, proliferation, and angiogenesis (Fig .1A) (10). Arsenic trioxide (ATO) targets various cellular functions through multiple molecular factors (Fig .1B). ATO plays dual roles in acute promyelocytic leukemia (APL) cells, and at low concentrations, it activates differentiation while at high concentrations, it promotes apoptosis (11). The aim of the present study was to appraise the combination effect of ATO and sorafenib on *VEGFA, B-RAF, MEK1, MEK2, Beclin1, LC3-II, ULK1, RAF1, BCL-2* and *PTEN* gene expression and apoptosis in leukemic cell lines.

## Materials and Methods

### Proliferation assayProliferation

The antiproliferative activity of ATO (0.5-5 μM) and sorafenib (2-12 μM) was assessed using MTT assay at 24, 48 and 72 hours, to distinguish optimal conditions with maximum effects, in KG-1 and U937 cells. In order to determine the growth inhibitory effects of ATO and sorafenib, KG-1 and U937 cells were seeded into 96- well plates at a primary density of 5×10^3^ per well (100 µl). After that, cells were treated with ATO, sorafenib and their combinations for 24, 48 and 72 hours. Control cells were treated with 0.1% DMSO alone. The proliferation rate of cells was analyzed by MTT assay and results are expressed as proliferation rate.

### Reagents

In this *in vitro* experimental study, annexin-V-FITC apoptosis detection kit, 3-(4, 5-dimethylthiazol-2-yl)-2, 5- diphenyltetrazolium bromide (MTT), dimethylsulfoxide (DMSO) and diethyl pyrocarbonate (DEPC)-treated water were obtained from Sigma-Aldrich (St. Louis, MO), and sorafenib was purchased from Santa Cruz (Dallas, Texas). ATO was provided by Sigma-Aldrich, St. Louis, MO, and dissolved in distilled water. RPMI 1640 medium and fetal bovine serum (FBS) were purchased from Gibco, Carlsbad, CA. The cDNA synthesis kit was purchased from Takara Bio Inc. (Otsu, Japan). TRI pure (used as the isolation reagent) was obtained from Roche Applied Science (Germany).

### Cell lines and treatment

We purchased U937 and KG-1 cell lines from the National Cell Bank of Iran (Pasteur Institute, Iran). Cell lines were cultured and expanded in RPMI 1640 supplemented with 10 and 20% heat-inactivated FBS for U937 and KG-1 cell line, respectively, 100 IU/ ml penicillin and 100 μg/ml streptomycin. Cells were cultured in a CO_2_ incubator at 37˚C with 5% CO_2_ in a humidified atmosphere. Cells were seeded at 1×10^5^ cells/ mL. For treatment experiments, prior to each assay, 80- 90% confluent flask was centrifuged, the supernatant was discarded and each cell pellet was resuspended separately in 1-2 ml of media and completely pipetted to prevent cell clumping. Then, 10 μL of cell solution including cell and media, was pipetted and cells were counted. Afterward, the cells were treated with the selected concentrations.

### Apoptosis assay

To assess the percentage of apoptosis induced by the above-noted compounds, fluorescein-conjugated annexin-V (annexin-V-FITC) staining assay was accomplished based on the manufacturer’s protocol. We treated KG-1 and U937 cells with ATO (1.618 and 2 μM for KG-1 and 1 μM for U937) and sorafenib (7 μM for KG-1 and 5 μM for U937) and their combination for 48 hours. Data acquisition and analysis of apoptosis by a Becton Dickinson (BD, America) flow cytometer and percentage of the annexin-V^+^/PI^-^ cells was recorded; finally, we used flowJo program to analyze our data.

### Cell cycle analysis

Here, U937 and KG-1 cell population were treated with specific concentrations of ATO and sorafenib for 48 hours, then fixed in cold 70% ethanol and stained with propidium iodide (PI). Cells were evaluated by BD flow cytometer instrument and data were analyzed by flowJo program. The apoptotic cell fraction was calculated based on hypodiploid G0/G1 DNA fraction.

### RNA isolation and real-time polymerase chain reaction

We treated KG-1 and U937 cells with ATO (1.618 and 2 μM for KG-1 and 1 μM for U937) and sorafenib (7 μM for KG-1 and 5 μM for U937) and their combination for 48 hours. Treated cells were harvested and dissolved in 1 ml of TRI pure (Roche Applied Science, Germany), based on the manufacturer’s instructions. DEPCtreated water was used to reconstitute the RNA pellets. The quantity and quality of total RNA were analyzed spectrophotometrically using Nanodrop ND-1000 (Nanodrop Technologies, Wilmington, DE) at 260 and 280 nm. Then, complementary DNAs (cDNAs) were reverse transcribed from 1-2 µg of total RNA by use of a cDNA synthesis kit (Takara Bio Inc., Japan) according to the manufacturer’s instructions. The concentration of cDNA was normalized in series of PCR through using *HPRT* and *GAPDH* primers. The normalized cDNAs were subjected to amplification, using Step One Plus™ ABI instrument (Applied Biosystems, USA). The levels of *HPRT* mRNA expression were used to evaluate the relative expression levels of the genes. The comparative Ct method was used to compute relative expression values. The primers and their corresponding amplicon lengths are provided in Table 1.

### Statistical analysis

Data were analyzed using GraphPad Prism 5 software by using one/two way ANOVA and for post-test evaluations, we used t test. All data represent the results obtained from triplicate independent experiments and expressed as mean ± standard errors of the mean (SE). Asterisks (*, **, and ***) in the Figures indicate P<0.05, P<0.01, and P<0.001, respectively.

**Table 1:** Real-time polymerase chain reaction primer


Gene	Primer sequence (5ˊ-3ˊ)	Reference

*GAPDH*	F: TGAACGGGAAGCTCACTGG	(12)
	R: TCCACCACCCTGTTGCTGTA	
*HPRT*	F: GCTATAAATTCTTTGCTGACCTGCTG	(13)
	R: AATTACTTTTATGTCCCCTGTTGACTGG	
*VEGFA*	F: AGGGCAGAATCATCACGAAGT	(14)
	R: AGGGTCTCGATTGGATGGCA	
*VEGFB*	F: GAGATGTCCCTGGAAGAACACA	(15)
	R: GAGTGGGATGGGTGATGTCAG	
VEGFC	F: GAGGAGCAGTTACGGTCTGTG	(16)
	R: TCCTTTCCTTAGCTGACACTTGT	
*VEGF-R1*	F: CAGGCCCAGTTTCTGCCATT	(14)
	R: TTCCAGCTCAGCGTGGTCGTA	
*VEGF-R2*	F: CCAGCAAAAGCAGGGAGTCTGT	(14)
	R: TGTCTGTGTCATCGGAGTGATATCC	
*LC3-II*	F: GATGTCCGACTTATTCGAGAGC	(17)
	R: TTGAGCTGTAAGCGCCTTCTA	
*Beclin1*	F: AGCTGCCGTTATACTGTTCTG	(17)
	R: ACTGCCTCCTGTGTCTTCAATCTT	
*ULK1*	F: TCGAGTTCTCCCGCAAGG	(18)
	R: CGTCTGAGACTTGGCGAGGT	
*BCL-2*	F: CTGCACCTGACGCCCTTCACC	(19)
	R: CACATGACCCCACCGAACTCAAAGA	
*PTEN*	F: TGGATTCGACTTAGACTTGACCT	(13)
	R: TTTGGCGGTGTCATAATGTCTT	
*AKT*	F: AGCGACGTGGCTATTGTGAAG	(13)
	R: GTACTCCCCTCGTTTGTGCAG	
*mTOR*	F: AACTCCGAGAGATGAGTCAAGA	(13)
	R: AGTTGGTCATAGAAGCGAGTAGA	
*PI3K*	F: AACACAGAAGACCAATACTC	(20)
	R: TTCGCCATCTACCACTAC	
*B-RAF*	F: CTCGAGTGATGATTGGGAGATTCCTGATGG	(21)
	R: CTGCTGAGGTGTAGGTGCTGTCAC	
*RAF-1*	F: CAG CCC TGT CCA GTA GC	(21)
	R: GCG TGA CTT TAC TGT TGC	
*MEK1*	F: ACCAGCCCAGCACACCAA	(22)
	R: GGGACTCGCTCTTTGTTGCTT	
*MEK2*	F: TGCTCACAAACCACACCTTCA	(22)
	R: ACACAACCAGCCGGCAAA	


## Results

### Evaluation of cell proliferation using MTT test

Metabolic activity can be detected through measuring the activity of succinate dehydrogenase as a mitochondrial enzyme via MTT assay. We applied the MTT assay to determine the anti-proliferative activity of ATO and sorafenib (alone and in combination) in U937 and KG-1 cell lines.

**Fig 2:**
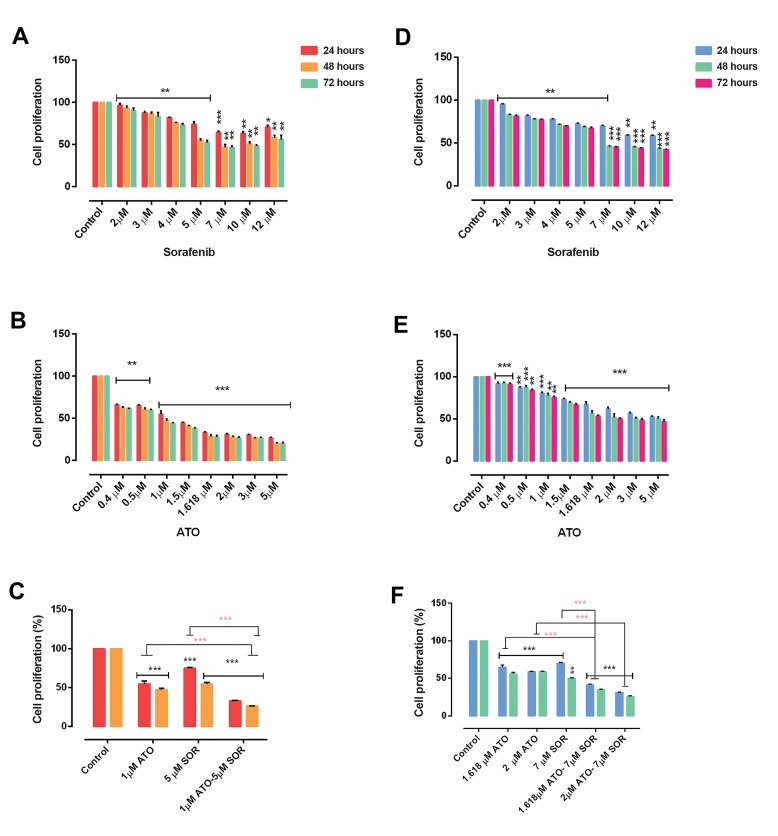
U937 and KG-1 cells proliferation. In U937 **A.** The anti-proliferative effects of sorafenib, **B.** Arsenic trioxide (ATO) and **C.** Their combinations. In KG-1** D.** The anti-proliferative effects of sorafenib,** E.** ATO, and **F.** Their combinations were assessed by MTT assay after 24, 48, and 72 hours treatment. Combination of ATO and sorafenib compared to the control or each compound alone, could significantly decrease cell proliferation in both cell lines. Data are expressed as mean ± SE of three independent experiments. Statistical significance was defined at *; P<0.05, **; P<0.01 and ***; P<0.001 compared to corresponding control and red star compared to combination therapy, by using two way ANOVA and t test.

We perceived both time- and dose-dependent effect of compounds. As seen in Figure 2, we did not see a significant difference between 48 and 72 hours treatment as assessed by two way ANOVA. Our data indicated that combination effect of ATO and sorafenib (P<0.001 for both cell lines) compared to the control or even singlecompound treatment (P<0.001 for KG-1 and P<0.01 for U937), could significantly decrease cell proliferation at 48 hours in both U937 and KG-1 cell lines (Fig .2).

### Apoptosis assay

**Fig 3:**
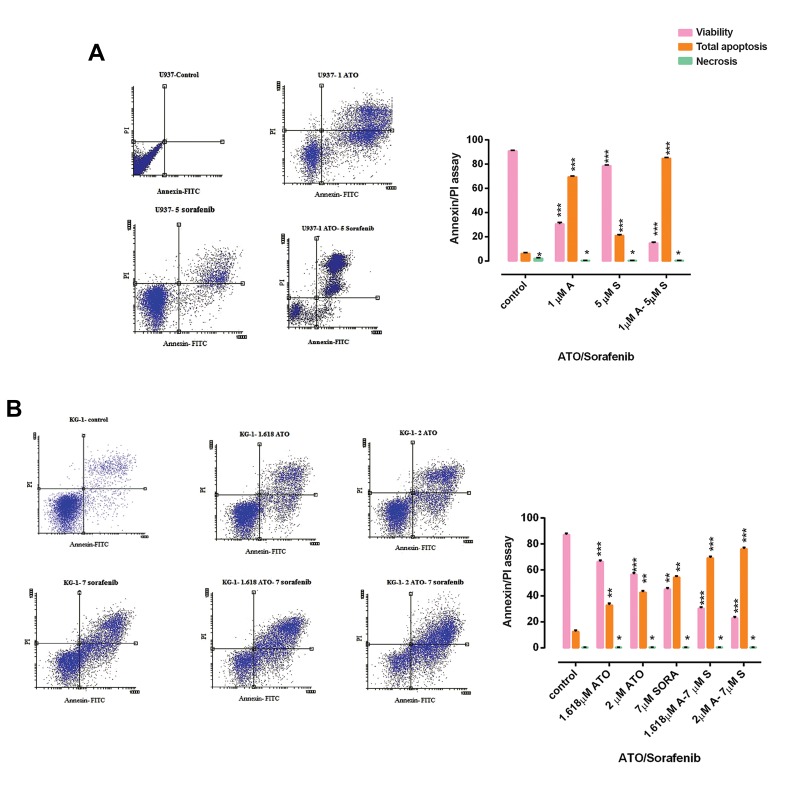
The rate of apoptosis and necrosis by flow cytometry. Investigation of apoptosis in **A.** U937 and **B.** KG-1 cell lines after 48 hours. Cells in the lower right quadrant show apoptosis while in the upper right quadrant show post-apoptotic necrosis. Data are expressed as mean ± SE of three independent experiments. Statistical significance was defined at *; P<0.05, **; P<0.01 and ***; P<0.001 compared to corresponding control, by using two way ANOVA.

To investigate apoptosis and necrosis, we performed flow cytometry assay using annexin-V FITC/PI staining for both U937 and KG-1 cell lines following 48h treatment. As seen in Figures 3A and B, our result indicated an increase in apoptotic cells (annexin+/PI) and minimum percentage of necrosis in treated cells compared to control, in both U937 and KG-1 cells. Moreover, we observed a significant increase (up to 70% in KG-1 and around 80% in U937 cells) in combination doses (P<0.001). The percentages of apoptotic cells in treated KG-1 and U937 cell lines were significantly higher than those of the control groups.

### Cell cycle assay

**Fig 4:**
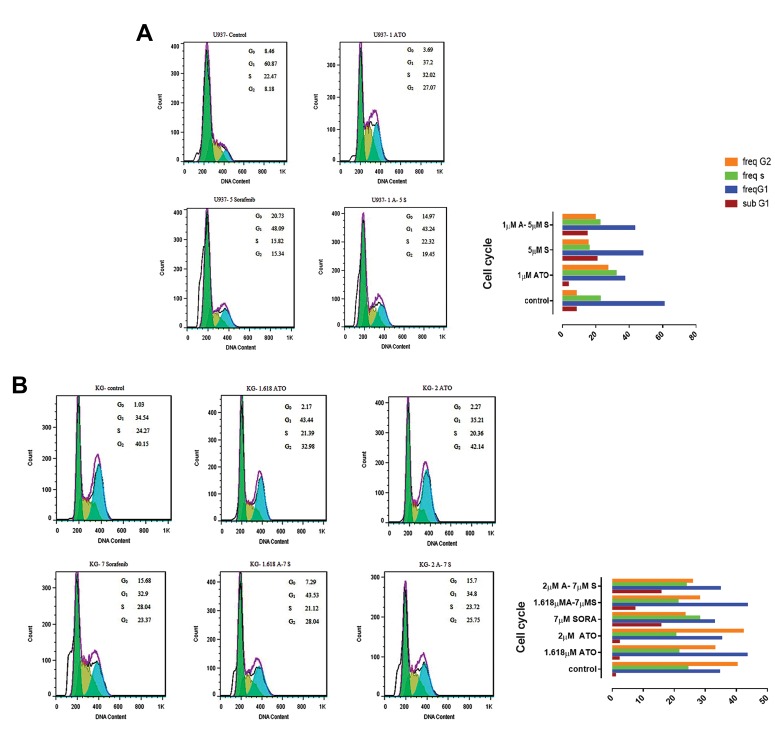
Cell cycle analysis. **A.** Cell cycle analysis for U937. Combination of arsenic trioxide (ATO) and sorafenib increased sub G1. **B.** Cell cycle analysis for KG-1. Effect of ATO and sorafenib on KG-1 increased sub-G0/G1 DNA population.

DNA content of U937 and KG-1 cells was assessed by flow cytometry. To specify the apoptosis activating role of ATO and sorafenib, U937 and KG-1 cells were treated with chosen doses for 48 hours. Our result indicated that combination of ATO and sorafenib increased hypodiploid G0/G1 DNA fraction in a dose-dependent manner (1.13 to 8.3% for KG-1 cell and 9.21 to 16.1% for U937 cell) (Fig .4).

### Real-time polymerase chain reaction assay

In order to investigate the mechanisms underlying the synergy observed for ATO and sorafenib, we analyzed gene expression of *B-RAF, MEK1, MEK2, Beclin1, LC3- II, ULK1, RAF1, BCL-2, PTEN, PI3K, AKT, mTOR,* and *VEGF* isoforms and its receptors (*VEGFR1* and *VEGFR2*) by real-time PCR.

**Fig 5:**
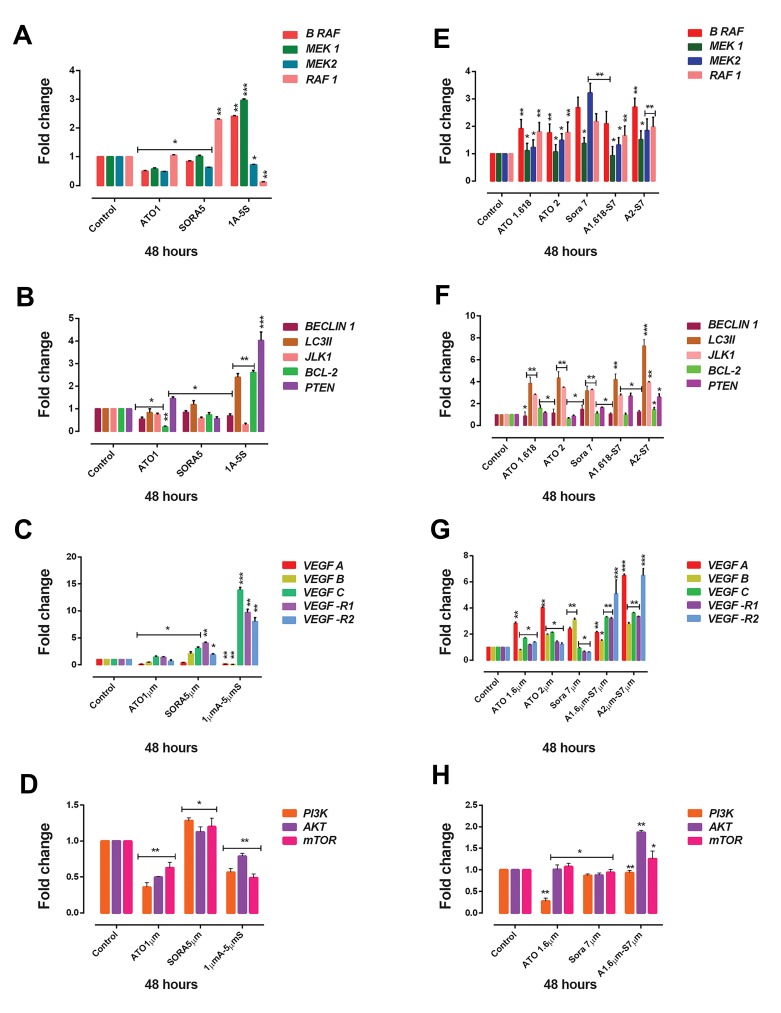
The effects of arsenic trioxide (ATO) and sorafenib on the mRNA level of indicated genes in U937 and KG-1 cells. In U937 cell line **A.** The effects of ATO and sorafenib on expression levels of cell proliferation genes, **B.** Autophagy genes, **C.** VEGF, **D.** Cell survival genes, and in KG-1 cells, **E.** The effects of ATO and sorafenib on expression levels of cell proliferation genes, **F.** Autophagy genes, **G.** VEGF, and **H.** Cell survival genes, were determined by realtime polymerase chain reaction (PCR) analysis. Values are given as mean ± SE of three independent experiments. Statistical significance was defined at *; P<0.05, **; P<0.01, ***; P<0.001 compared to corresponding control by using two way ANOVA and t test, and VEGF: Vascular endothelial growth factor.

U937 cells were treated with specific concentrations of ATO (1 μM), sorafenib (5 μM) and their combination for 48 hours. We observed that the expression of *B-RAF* and *MEK1* decreased when cells were treated with a single compound (P<0.05) while increased when treated with the combination dose (P<0.001) in comparison with the control. But, the expression of *MEK2* decreased following treatment with chosen doses (both single and combination) (P<0.05). Moreover, in this pathway, the expression of RAF1 was markedly decreased following treatment with the combination dose (P<0.01). Furthermore, the expression of *BCL-2* decreased while cells were treated with a single compound (P<0.05), but slightly increased following treatment with the combination dose (P<0.01). The expression ofPTENas a tumor suppressor significantly increased after treatment with the combination dose. In addition, expression of *PI3/AKT/mTOR* decreased following treatment with the combination dose (P<0.001). Among the autophagy-related genes, we observed that the level of expression of *ULK1* (P<0.01) and *Beclin1* (P<0.05) decreased after combination treatment while the expression of *LC3-II* increased (P<0.01) following treatment with the combination dose (Fig .5A-D).

KG-1 cells were treated with ATO (1.618 and 2 μM), sorafenib (7 μM) and their combination for 48 hours. Our data indicated that the expression of *B-RAF* (P<0.001), *MEK1* (P<0.05), *MEK2* (P<0.001), and RAF1 (P<0.001) increased following treatment with the combination doses. Furthermore, the expression of *BCL-2* slightly increased (P<0.05) following treatment with the combination doses. The expression of *PTEN* significantly increased after treatment with combination dose (P<0.05). Moreover, the expression of *AKT* (P<0.01) and *mTOR* (P<0.05) slightly increased following treatment with the combination of ATO and sorafenib. In addition, the expression of Beclin1 (P<0.05), *LC3-II* (P<0.001 for the combination of ATO 2 μM and sorafenib 7 μM) and / (P<0.01 for the combination of ATO 2 μM and sorafenib 7 μM) as autophagy activators, increased in KG-1 cells. Since autophagy signaling pathway plays a dual role in cancer cells, activation of this pathway may promote programmed cell death (Fig .5E-H).

## Discussion

In the present research, we tried to assess the *in vitro* activity of sorafenib and ATO, alone and in combination, in AML cell lines. AML is known as a heterogeneous disorder. Despite advanced treatment options which have to promote overall survival, AML still remains as a lifethreating disease (23). In the current article, we studied the effect of ATO and sorafenib on the expression pattern of *VEGFA* (24), *B-RAF, MEK1, MEK2, Beclin1, LC3-II, ULK1, RAF1, BCL-2 *and *PTEN* in leukemic cell lines. We focused not only on apoptosis but also on autophagy. Previous studies demonstrated that angiogenesis factors such as VEGF-A play a vital role in cancer progression and metastasis (25). Autophagy is a major protein degradation process that contributes to maitainence of intercellular hemostasis (26). The critical and dual role of autophagy has been confirmed in various studies. Any dysfunction of this pathway may contribute to cancer progression, and metastasis or drug resistance.

ATO as a multi-target agent is able to activate apoptosis and autophagy (27) through various molecular pathways in numerous cancers including solid tumor cells and hematological malignancies. In this study, we observed ATO cytotoxic and apoptosis-inducing effects in both U937 and KG-1 cell lines in a dose and time-dependent manner. Our data indicated that ATO can influence cell proliferation and cell death pathway. We examined a wide range of ATO concentrations in both resistant and sensitive cell lines. We observed that 1.618 and 2 μM of ATO has a significant effect as compared to its lower concentrations in KG-1 (as a resistant cell line). Chiu et al. (28) reported that ATO in combination with ionizing radiation may enhance programmed cell death by activating both autophagy and apoptosis in human fibrosarcoma cells. Also, Chiu et al. (29) confirmed that ATO can synergistically activate both apoptosis and autophagy.

Sorafenib is known as a multikinase inhibitor which act through suppression of Ser/Thr kinase Raf that is known to have an important role in tumor cell signaling and proliferation, and various RTKs involved in angiogenesis, such as VEGF (30). However, sorafenib was shown to be more effective in leukemia with the FLT3-ITD mutation, and its antileukemic function was clarified in several patients with AML and wild-type form of FLT3 (31). In our previous study, we demonstrated that sorafenib downregulates the gene expression of *VEGFR-1/2* in KG-1 cell line and downregulates the gene expression of *VEGF-A* in *U937* cell line (32).

The RAF/MEK/ERK signaling pathway was shown to be activated in various processes in cancer. In the present study, we observed that the expression of B-RAF, MEK1, MEK2, and RAF1 increased as a result of treatment with ATO, sorafenib and their combination in KG-1 cell line. In addition, the expression level of *MEK2, RAF1, Beclin1, ULK1, VEGFA* and *VEGFB* decreased following treatment with the combination dose in U937 cell line while the expression of *LC3-II* incresed. Various studies reported that blockade of the MEK/ERK pathway by ATO treatment, induces apoptotic cell death. Fecteau et al. (33) reported that sorafenib downregulates VEGFR and the RAF/MEK/ERK signaling pathways.

We observed that the expression of *Beclin1, LC3-II* and *ULK1* as autophagy activators increased following treatment with ATO, sorafenib and their combination in KG-1 cell line. Our data indicated increases in the expression of *LC3-II* and *PTEN* which may lead to activation of both autophagy and apoptosis. Consistent with our result, a group of scientists reported that ATO decreased the gene expression level of *Beclin1, LC3-II* and *MAPK* signaling pathways in U118-MG cells (29). Li et al. (34) by studying Beclin 1 and LC3Ⅱ, indicated that inhibiting autophagy promotes the cytotoxic effect of ATO in glioblastoma cells. Goussetis et al. (35) reported that ATO can activate autophagy in the leukemic cells; induction of autophagy process seems to involve activation of the ERK pathway. Chiu et al. (36) demonstrated that ATO in combination with ionizing radiation, could initiate autophagy through activation of ERK and inhibition of PI3K/AKT signaling pathway. Wang et al. (37) showed that mice xenografted with FLT3-ITD MOLM13 cell line and treated with a combination of sorafenib and ATO have remarkably promoted survival. This combination has the potential to prosper the therapeutic effect of FLT3-ITD in patients with AML.

Tai et al. (38) reported that sorafenib-induced autophagy signaling pathway through significant induction of LC3-II in HCC cell lines. Shimizu et al. (39) demonstrated increased expression of *LC3-II* which led to autophagosome formation and autophagy activation while expression of Beclin 1 did not change under sorafenib treatment. Amantini et al. (40) using bladder cancer cells, reported that sorafenib induces apoptosis through blocking Akt and activating PTEN.

## Conclusion

In this study, we found that combination of ATO and sorafenib significantly reduced the viability of U937 and KG-1 cells. In addition, the crosstalk between apoptosis and autophagy is complicated and varies among different cell types. Similar stimuli may activate both pathways as they share various signaling. ATO with antileukemic activity in AML cell lines, enhances the antitumor activity of sorafenib in both U937 and KG-1 cells. Our study indicated a potential mechanism underlying the interaction between ATO and sorafenib in U937 and KG-1 cell lines.
